# A Novel Deltaflexivirus that Infects the Plant Fungal Pathogen, *Sclerotinia sclerotiorum*, Can Be Transmitted Among Host Vegetative Incompatible Strains

**DOI:** 10.3390/v10060295

**Published:** 2018-05-31

**Authors:** Muhammad Rizwan Hamid, Jiatao Xie, Songsong Wu, Shahzeen Kanwal Maria, Dan Zheng, Abdoulaye Assane Hamidou, Qihua Wang, Jiasen Cheng, Yanping Fu, Daohong Jiang

**Affiliations:** 1 State Key Laboratory of Agriculture Microbiology, Huazhong Agricultural University, Wuhan 430070, China; rizwan.phyto@outlook.com (M.R.H.); jiataoxie@mail.hzau.edu.cn (J.X.); wusong425@126.com (S.W.); shehzeen.kanwal@outlook.com (S.K.M.); zd1991st@163.com (D.Z.); doul_as@yahoo.fr (A.A.H.); wqhtzyy@163.com (Q.W.); jiasencheng@mail.hzau.edu.cn (J.C.); yanpingfu@mail.hzau.edu.cn (Y.F.); 2 Provincial Key Laboratory of Plant Pathology of Hubei Province, College of Plant Science and Technology, Huazhong Agricultural University, Wuhan 430070, China

**Keywords:** *Sclerotinia sclerotiorum*, *Brassica napus*, deltaflexivirus, transmission, vegetative incompatibility

## Abstract

Various mycoviruses have been isolated from *Sclerotinia sclerotiorum*. Here, we identified a viral RNA sequence contig, representing a novel virus, Sclerotinia sclerotiorum deltaflexivirus 2 (SsDFV2), from an RNA_Seq database. We found that SsDFV2 was harbored in the hypovirulent strain, 228, which grew slowly on potato dextrose agar, produced a few sclerotia, and could not induce typical lesions on detached rapeseed (*Brassica napus*) leaves. Strain 228 was also infected by Botrytis porri RNA Virus 1 (BpRV1), a virus originally isolated from Botrytis porri. The genome of SsDFV2 comprised 6711 nucleotides, excluding the poly (A) tail, and contained a single large predicted open reading frame encoding a putative viral RNA replicase. Phylogenetic analysis demonstrated that SsDFV2 is closely related to viruses in the family *Deltaflexiviridae*; however, it also differs significantly from members of this family, suggesting that it may represent a new species. Further we determined that SsDFV2 could be efficiently transmitted to host vegetative incompatible individuals by dual culture. To our best knowledge, this is the first report that a (+) ssRNA mycovirus can overcome the transmission limitations of the vegetative incompatibility system, a phenomenon that may facilitate the potential use of mycoviruses for the control of crop fungal diseases.

## 1. Introduction

*Sclerotinia sclerotiorum* (Lib.) de Bary, an ascomycetous fungus, is a devastating necrotrophic plant pathogen with wide host range that encompasses economically important crops, including vegetable crops, and is responsible for heavy global agricultural losses each year [1]. The control of diseases caused by *S. sclerotiorum* depends mainly on chemical fungicides, since no resistant cultivars are available. Therefore, it is important to explore novel, environmentally friendly alternatives to chemical fungicides or to reduce amounts of chemical fungicides used.

Mycoviruses replicate in fungal cells and are widespread in nature. Among plant fungal pathogens, some mycoviruses can confer hypovirulence to their hosts and, therefore, have potential as biological agents for disease control [2,3,4]. Mycoviruses can be transmitted vertically, via asexual reproduction (or rarely via sexual reproduction) and horizontally, via hyphal anastomosis, which is controlled by the vegetative incompatibility system that inhibits hyphal fusion [5]. The fact that mycoviruses cannot be efficiently transmitted among vegetative incompatibility groups severely restricts the use of hypovirulence-associated mycoviruses for the control of fungal diseases [6]. The use of hypovirus, a mycovirus that infects the chestnut blight fungus, *Cryphonectria parasitica*, to control chestnut blight in European countries has become a textbook example of the biological control of plant fungal diseases [7,8,9]; however, this method is also subject to the restrictions of vegetative incompatibility. More recently, genetically modified strains of *C. parasitica* that can function as super donors for hypovirus transmission have been constructed. These engineered strains overcome the limitations of hypovirus transmission caused by host vegetative incompatibility and have clear potential for use in control of chestnut blight in forests [8]. However, this strategy is not likely to function in other fungi with more complex vegetative incompatibility, or where genes associated with vegetative incompatibility are involved in other biological traits of fungi (such as growth, conidiation, and stress tolerance). Previously, we identified a DNA virus that could be transmitted among host vegetative incompatible groups and used insects as a transmission vector [10,11], and a mycoreovirus that could suppress host vegetative incompatibility responses [12], suggesting that vegetative incompatibility may not be an insurmountable barrier for some specific mycoviruses. Screening for mycoviruses that can be efficiently transmitted among vegetative incompatible host groups is a potential way to overcome the limitations of the use of mycoviruses to control fungal diseases.

Numerous mycoviruses from *S. sclerotiorum* have been identified, including viruses from the families, Hypoviridae, Alphaflexiviridae, Endornaviridae, Genomoviridae, Mymonaviridae, Narnaviridae, Reoviridae, Megabirnaviridae, Narnaviridae, and the new families such as Deltaflexiviridae and the proposed Fusariviridae [12,13,14,15,16,17,18,19,20,21]. Furthermore, novel viruses have been successfully detected through analysis of novel viral sequences obtained by metatranscriptomic analysis of *S. sclerotiorum* strains isolated from Australia [22]. Together, these discoveries suggest that the mycoviruses that infect *S. sclerotiorum* are varied and widespread, and may provide new insights into virus taxonomy and ecology [18,23,24].

To identify novel viruses with potential to control fungal diseases, we initially collected a total of 173 abnormal strains of *S. sclerotiorum* from diseased rapeseed (*Brassica napus*) plants in China, and performed RNA-sequencing analysis of pooled samples. We found that one contig (contig 1178), with an incomplete ORF encoding a putative RNA-dependent RNA Polymerase (RdRp), had similarities with a previously reported Soybean leaf-associated mycoflexivirus 1 (SlaMFV1) in the RNA-sequencing database [21]. Furthermore, a contig with 397 nt in length (Ss-AA_clean.1_(paired)_contig_15254) whose sequence was 94.5% identity to the virus represented by contig 1178 was also obtained in the RNA_sequence database for Australian strains of *S. sclerotiorum* [22]. The presence of this contig suggests the existence of a novel mycovirus in *S. sclerotiorum* that is phylogenetically related to flexivirus. Further RT-PCR analysis indicated that strain 228 carries this novel flexivirus. In this study, we identified and characterized this novel virus and investigated its properties, including its impact on virulence and transmission among strains of *S. sclerotiorum*.

## 2. Materials and Methods

### 2.1. Fungal Strains and Culture

*S. sclerotiorum* strain 228 was isolated from a sclerotium collected from diseased rapeseed in a field in Hubei province, PR China. Strains N36, Ep-1PNA367, and 1980, were fused as reference control strains in this study. Virus-infected strain 228 and virus-free strains N36, Ep-1PNA367, and 1980 were grown on laboratory-produced potato dextrose agar (PDA) at 20–22 °C, and stored on PDA slants at 4–6 °C. Sclerotia were dried at room temperature and maintained at −20 °C for long term conservation. In this study, strains Ep-1PNA367 and 1980 incorporating a hygromycin-resistance gene (Ep-1PNA367*^hph^* and 1980*^hph^*) were used to determine virus transmission between vegetative incompatible strains. Virus was introduced into strains Ep-1PNA367 and 1980 by dual culture; the resulting virus-containing strains were named Ep-1PNA367VI and 1980VI.

### 2.2. Analysis of the Biological Properties of Strain 228

The biological properties of strain 228, including colony morphology, growth rate, and virulence were compared to these of strain N36, as described previously by Xie et al., 2009 [25]. The colony morphology of both strains on PDA medium was observed every 24 h, and photographs taken 7 days after inoculation. Daily radial growth rates were recorded and calculated using a ruler 3–5 times at intervals of 24–48 h and repeated three times. For the pathogenicity test, actively growing mycelial agar discs from both virus-containing and virus-free strains were inoculated on detached rapeseed leaves. Inoculated leaves were then incubated in a 20 °C incubator for 72 h. Growth rate data were subjected to analysis of variance (ANOVA) using the Statistix 8.0 program (Analytical Software, Tallahassee, FL, USA).

### 2.3. Extraction and Purification of dsRNA

Double-stranded RNA (dsRNA) was extracted and purified from strain 228 cultured on cellophane membranes laid on PDA plates (9 cm diameter). Fresh mycelia were harvested by roiling the colony on cellophane membrane using a sterile blade 2–3 days post-inoculation and ground into a fine powder using liquid nitrogen and a mortar and pestle. dsRNA extraction in the presence of cellulose (Sigma-Aldrich, Dorset, UK) was performed by chromatography as previously described in detail by Xie et al., 2015 [26]. Extracted dsRNA samples were treated with DNase 1 and S1 nuclease (Takara, Dalian, China). Then, treated and non-treated individual dsRNA segments were re-isolated and purified from 1% agarose gels using a gel extraction kit (Axygen Scientific Inc., Wujiang, China) and stored at −80 °C for further use.

### 2.4. Confirmation of Putative Mycoviruses

An RNA_Seq database of 173 strains of *S. sclerotiorum* was constructed and viral genome contigs identified by comparison with the NCBI viral reference amino acid sequence database using USEARCH [27]. Among viral contigs, one contig (contig 1178) of 5667 bp, representing a novel virus with 35% identity to the sequence of SlaMFV1, and another (First_contig 20) of 2895 bp whose sequence was almost identical to the Botrytis porri RNA Virus 1 (BpRV1), were selected for further analysis. To detect and verify the presence of the novel virus and BpRV1, RNA samples extracted from individual strains were evaluated by RT-PCR amplification using specific PCR primers for the novel virus and BpRV1 (Table 1), designed based on the contig sequences. Total RNA samples obtained from fresh mycelia were used as template for cDNA synthesis using a cDNA synthesis kit (TaKaRa, Dalian, China) and tagged random dN6 primers (Table 1). After reverse transcription, the resulting cDNA samples were used as input material for PCR amplification to detect mycoviral RdRp sequences. For each RT-PCR, reaction mixtures were incubated at 50 °C for 30 min, then 94 °C for 2 min, followed by 32 cycles of 94 °C for 30 s, 55 °C for 30 s, and 72 °C for 45 s, using Ex Taq DNA polymerase (TaKaRa, Dalian, China). PCR products of the expected size were purified, sequenced, and analyzed using the DNAMAN (Lynnon Corporation, San Ramon, CA, USA) and BLASTX programs to search the NCBI database to determine whether sequences were mycoviral.

### 2.5. Rapid Amplification of cDNA Ends (RACE) and Sequencing

To obtain a complete cDNA sequence of the purified dsRNA segment, the PC3-T7 loop adapter (Table 1) was ligated to the 5′ end and 3′ of the purified dsRNA, as previously described by Darissa et al., 2010 [28]. The dsRNA ligated to the PC3-T7 loop was purified using a nucleic acid purification kit (Axygen Scientific Inc., Wujiang, China), denatured in DMSO, and subsequently reverse transcribed. The cDNA sequences of the 5′- and 3′-termini were determined by RACE, using the primer pairs listed in Table 1. The PCR products generated were then separated by gel electrophoresis, purified using a gel extraction kit, cloned into the vector, pMD18-T, and sequenced. All of the amplicons obtained in the present study were sequenced by Beijing Tianyi Biotechnology Co., Ltd. (Beijing, China).

### 2.6. Bioinformatics and Sequence Analysis

A motif search was conducted using Transfac (http://www.genome.jp/tools/motif/). Multiple sequence alignments were performed using CLUSTALW (http://www.genome.jp/tools-bin/clustalw) [29]. Vector sequences were removed from sequencing data using the NCBI VecSec tool (https://www.ncbi.nlm.nih.gov/tools/ vecscreen/). All phylogenetics trees were constructed using the neighbor-joining method using MEGA version 7.0 [30]. Phylogenetic trees were first plotted using TreeView and then edited manually.

### 2.7. Viral Transmission Assay

To assess the possibility of horizontal transmission of SsDFV2 between strains of *S. sclerotiorum*, fresh mycelial agar discs from strains 228 and Ep-1PNA367*^hph^*, or 228 and 1980*^hph^*, were dual-cultured on PDA plates. After 7 days of dual culture, mycelial agar discs from the growth side of strain Ep-1PNA367*^hph^* were transferred to fresh hygromycin-containing PDA plates, and then to fresh PDA plates overlaid with cellophane for further incubation for 3 days before viral detection [13]. To determine whether SsDFV2 or BpRV1 could be transmitted from strain 228 to its vegetative incompatible strain, Ep-1PNA367, strains 228 and Ep-1PNA367*^hph^* were dual cultured on PDA for 7 days, and then 10 mycelial agar discs from the growth side of strain Ep-1PNA367*^hph^* were transferred to a fresh hygromycin-containing PDA plate (Figure S1a,b), followed by transfer to fresh PDA plates for virus determination. Furthermore, to understand the independent transmission of SsDFV2 among strains, strain Ep-1PNA367VI (only infected by SsDFV2) and strain 1980*^hph^* were dual-cultured on PDA medium. Mycelial agar discs were taken from 1980*^hph^* colonies, seeded on hygromycin-containing PDA plates, and the mycelial mass harvested from newly developed colonies for virus determination.

## 3. Results

### 3.1. Strain 228 is a Hypovirulent Strain of S. sclerotiorum Co-Infected by Two Viruses

**Figure 1:**
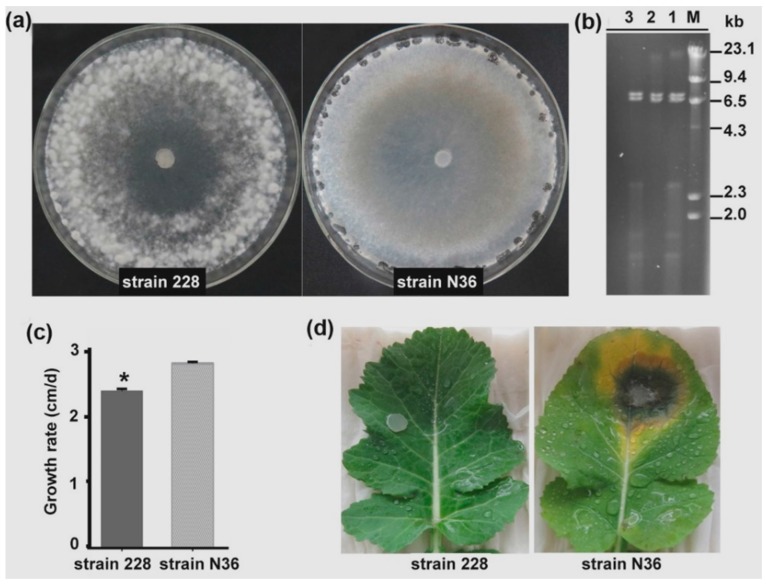
Biological characteristics of *S. sclerotiorum* strain 228 and its dsRNA elements. (**a**) Colony morphology of strains 228 and N36 (virus-free) grown on PDA for 7 days at 20 °C; (**b**) Double-stranded RNA elements in strain 228. Lane 1, untreated dsRNA; Lane 2, dsRNA treated with S1 nuclease; Lane 3, dsRNA treated with DNase I, prior to electrophoresis; (**c**) Growth rate of two *S. sclerotiorum* strains. Values that are significantly different (*p* < 0.05) are indicated by asterisks; (**d**) Assay of the virulence of strains 228 and N36 on detached rapeseed leaves at 72 h post-inoculation at 20 °C and 100% relative humidity.

RT-PCR amplification indicated that strain 228 may harbor a novel mycovirus, represented by contig 1178. Therefore, we selected strain 228 for further study. When grown on PDA, strain 228 formed an abnormal colony, with much richer aerial hyphae than a wild-type strain (N36) with a normal growth rate and virulence. Although strain 228 could form sclerotia given time, only a few sclerotia were produced from colonies at later stages (Figure 1a). Strain 228 also grew more slowly than strain N36, with a growth rate of 2.4 cm/day, while the growth rate of strain N36 was 2.82 cm/day (Figure 1c). Strain 228 exhibited very low virulence; it was unable to cause any clear lesion on leaves detached from rapeseed plants (Figure 1d).

**Figure 2:**
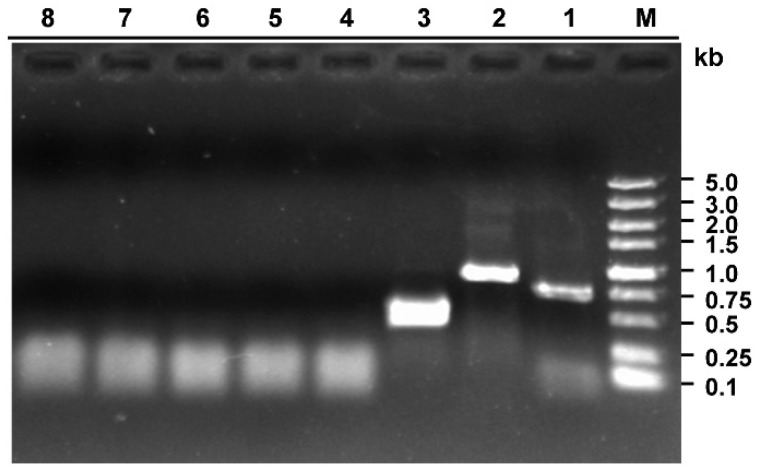
RT-PCR amplification confirming that *S. sclerotiorum* strain 228 harbored two viruses. Primer pairs were designed based on the sequences of contig 1178 and First_contig 20, representing a novel virus (SsDFV2) and a previously reported virus (BpRV1), respectively. Lane M, DNA weight marker; Lane 1, SsDFV2 specific band in strain 228; Lane 2, BpRV1 (dsRNA1) specific band in strain 228; Lane 3, BpRV1 (dsRNA2) specific band in strain 228; Lanes 4,5, Neither SsDFV2 nor and BpRV1 were detected in strain N36; Lanes 6,7, Neither SsDFV2 nor BpRV1 were detected in strain Ep-1PNA367; Lane 8, ddH_2_O control.

An RNA sample was extracted from strain 228 and subjected to RT-PCR amplification using primer pairs designed based on the sequence of contig 1178. RNA samples from strains N36 and Ep-1PNA367 were used as controls. As expected, a DNA fragment of approximately 750 bp was amplified from strain 228 cDNA, while no DNA fragment of the expected size was amplified from strain N36 and Ep-1PNA367 (Figure 2). Sequencing of the RT-PCR product demonstrated that it was 100% identical to that of contig 1178, confirming that strain 228 was infected by a novel virus, represented by contig 1178. We temporarily named this novel virus, *Sclerotinia sclerotiorum* deltaflexivirus 2 (SsDFV2).

dsRNA samples were extracted from strain 228, treated with DNase 1 and S1 nuclease to remove ssRNA and host genomic DNA, and separated by electrophoresis on a 1% agarose gel. Unexpectedly, the dsRNA sample contained at least two dsRNA fragments of very similar size, suggesting that strain 228 may harbor more than one mycovirus (Figure 1b). To determine which virus co-infected strain 228 along with SsDFV2, we designed additional specific primer pairs based on our previously constructed virus contigs. We identified an RT-PCR product of 1200 bp using specific primer pairs based on the sequence of First_contig 20 (Figure 2). This contig was 2895 bp in size and had 100% sequence identity with the dsRNA1 of *Botrytis porri* RNA virus 1 (BpRV1). As BpRV1 has two dsRNA fragments, the previously reported sequence of BpRV1 dsRNA2 in the NCBI database was used to design another primer pair, which was used to amplify an RT-PCR product of approximately 500 bp, confirming its presence in *S. sclerotiorum* strain 228 (Figure 2). These data suggest that strain 228 was also infected by BpRV1.

### 3.2. Genome Organization of Sclerotinia sclerotiorum Deltaflexivirus 2

**Figure 3:**
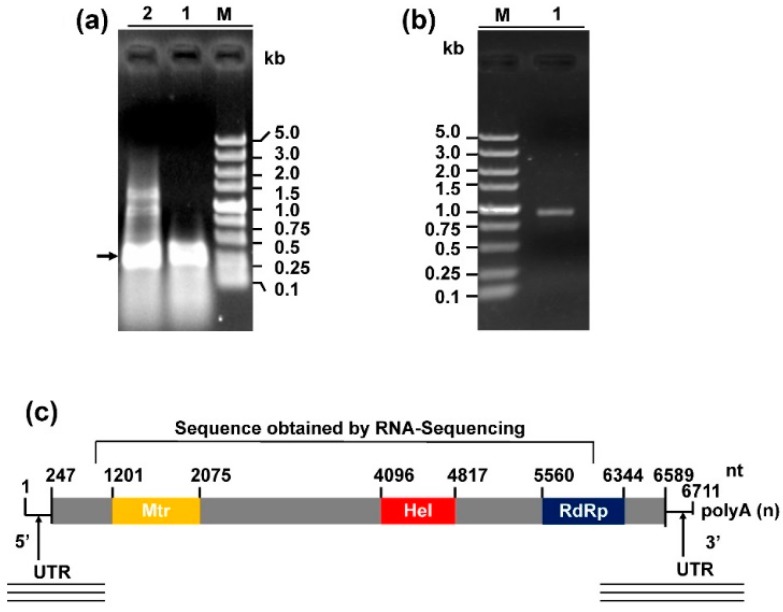
Cloning of the termini of the SsDFV2 genome and a schematic of the viral genome. (**a**) 5 RACE. Lane M, DL5000 DNA marker; Lane 1, band containing the 5 terminal sequence of SsDFV2 in strain 228; Lane 2, nested PCR producing more than one band. The bands of approximately 1.5, 1.0, and 0.45 kb were sequenced, and the 0.45 kb band (arrow) contained 5 terminal overlapping sequence, whereas the two larger bands were *S. sclerotiorum* genes. (**b**) 3 RACE. Lane M, DL5000 DNA Marker; Lane 1, band containing the 3 terminal sequence of SsDFV2, with a Poly-T tail, from strain 228. (**c**) Schematic representation of the genomic organization of SsDFV2. The open reading frame is represented by the box. Nucleotide numbers are indicated. Mtr, viral methyltransferase; Hel, helicase 1; RdRp_2, RNA dependent RNA polymerase, lines under the sketch represent sequenced RACE clones.

We obtained the 5- and 3-terminal sequences of SsDFV2 using the RACE technique (Figure 3a,b). Then, we assembled the two terminal sequences, together with the sequence of contig 1178 to generate the full-length genome sequence of SsFDV2. The full-cDNA sequence of SsDFV2 was deposited in the GenBank database under the accession number MH299810. The genome of SsDFV2 was composed of 6711 nucleotides (nt) excluding its poly (A) tail. It contained a 247 nt 5-UTR and a 122 nt 3-UTR (excluding the poly (A) tail) (Figure 3c). The GC content of the whole SsDFV2 genome was 56.2%. The genome was predicted to encode a single large open reading frame (ORF), beginning with an AUG codon at nt 245–247, and terminating with a UAA codon at nt 6587–6589. The ORF encoded a putative polyprotein of 2113 amino acids with a calculated molecular mass of 235 kDa and a predicted pI value of 9.1. A conserved motif search, conducted using Transfac (http://www.genome.jp/tools/motif/), revealed that the ORF-encoded protein contained three putative conserved domains: viral methyltransferase (Mtr), Viral (Superfamily 1) RNA helicase (Hel), and RNA dependent RNA polymerase (RdRp) (Figure 3). Thus, we conclude that this putative protein was the viral RNA replicase of SsFDV2.

### 3.3. SsDFV2 is Closely Phylogenetically Related to Flexiviruses

**Figure 4:**
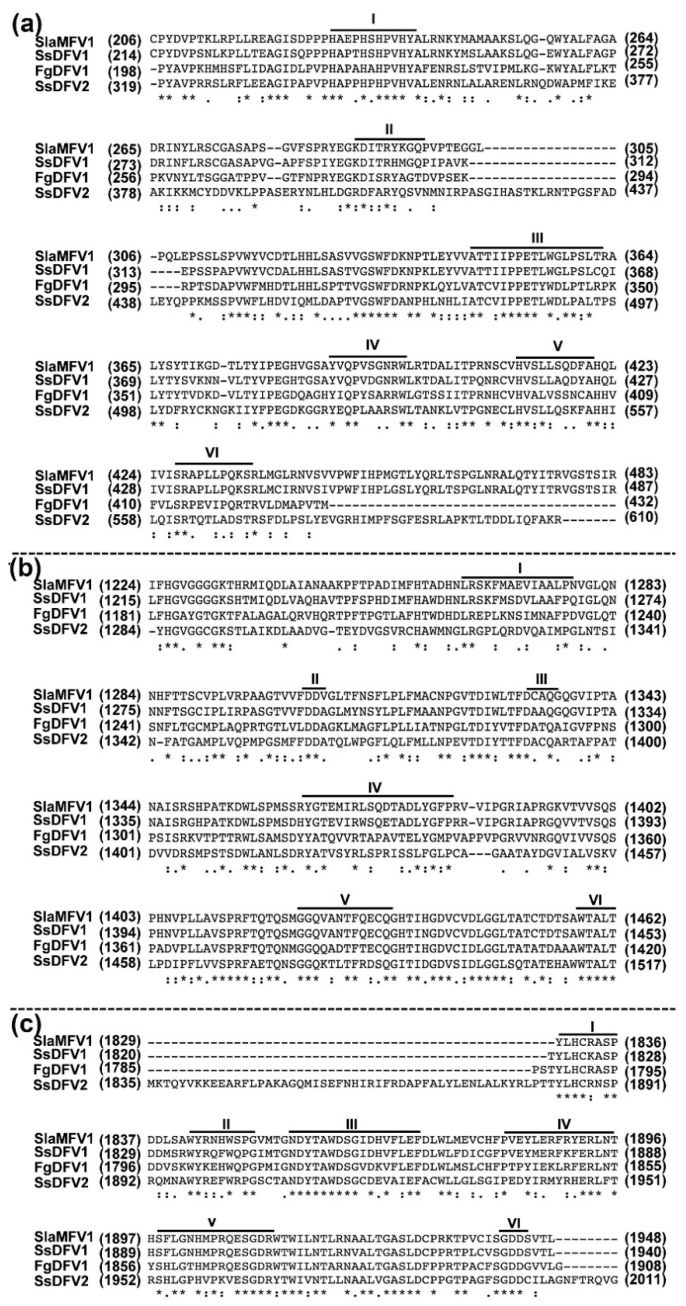
Multiple alignments of the three conserved domains of SsDFV2 and corresponding regions in reference members of the order Tymovirales. (**a**) methyltransferase; (**b**) helicase; (**c**) RdRp. Motif positions are indicated by lines above the sequences numbered from I to VI. Identical residues are indicated by asterisks, and conserved and semi-conserved amino acid residues by colons and dots, respectively. Numbers in brackets correspond to the number of amino acid residues separating the motifs. Abbreviations of virus names and sequence accession numbers are as indicated in Table 2.

Based on the sequence of contig 1178, SsDFV2 was predicted to be related to viruses of the family *Deltaflexiviridae*. We aligned the conserved domains (Mtr, Hel, and RdRp) of the putative RNA replicase of SsDFV2 with those of homologous domains from selected other viruses, using pairwise comparison to determine the percentage of amino acid sequence identity (Figure 4 and Table 2).

**Figure 5:**
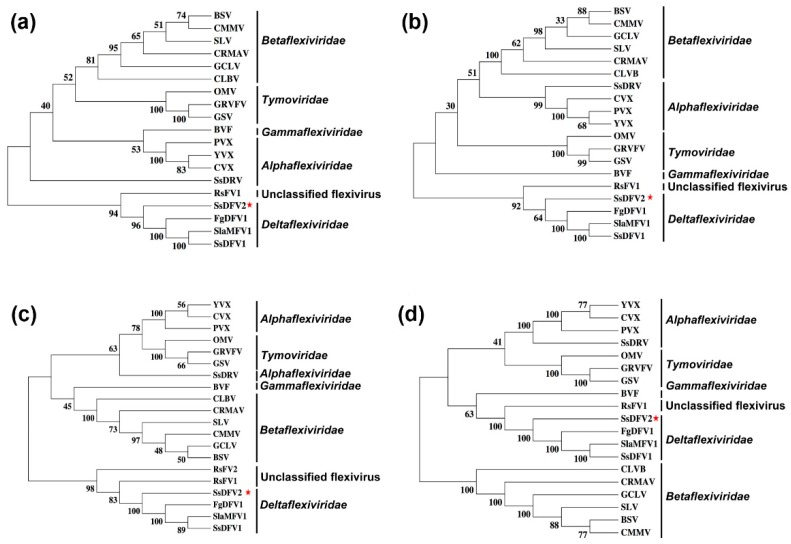
Phylogenetic analysis (Bootstrap concensus tree) of SsDFV2 and other selected viruses using the Neighbor joining method with 1000 bootstrap replications. Comparisons of conserved (**a**) helicase; (**b**) methyltransferase; (**c**) RdRp; and (**d**) the entire replication-associated polyprotein among members of several genera of the families *Alpha-*, *Beta-*, and *Gamma-*, *Deltaflexiviridae*, and unclassified tymoviruses. The data coverage percentage (support out of 1000 bootstrap replications) is shown by the numbers on the left of branches. The position of novel virus SsDFV2 is shown by red asterisks. Abbreviations of virus names and sequence accession numbers are as indicated in Table 2.

A putative methyltransferase domain was located close to the N-terminal region of the predicted RNA replicase. Multiple alignment revealed that this conserved domain contains six conserved motifs I–VI (Figure 4a), with 38% (E-value = 4 × 10^−57^), 38% (E-value = 3 × 10^−48^), and 35% (E-value = 3 × 10^−43^) sequence identity to the viral methyltransferase domains of Fusarium graminearum deltaflexivirus 1 (FgDFV1), SlaMFV1, and SsDFV1, respectively (Table 2). Phylogenetic analysis showed that the methyltransferase domain of SsDFV2 was clustered with those of viruses in the new family *Deltaflexiviridae* (Figure 5b).

The viral RNA helicase 1 (Helicase, Hel) domain homologous region within the putative RNA replicase of SsDFV2 showed significant sequence identity (approximately 13%–46%) with the Hel encoded by viruses from the order Tymovirales, with 46% (E-value = 6 × 10^−46^), 42% (E-value = 6 × 10^−48^) and 43% (E-value = 2 × 10^−51^) identity to those of FgDFV1, SlaMFV1, and SsDFV1, respectively (Table 2). Multiple alignments suggested that the helicase domain of SsDFV2 contains six viral RNA helicase 1 conserved motifs (I–VI) (Figure 4b).

The RdRp domain of SsDFV2 contains six typical viral RdRp motifs (Figure 4c) and exhibited significant sequence identity (approximately 16%–51%) with the RdRps encoded by viruses from the order Tymovirales (Table 2), and 51% (E-value = 1 × 10^−80^), 50% (E-value = 4 × 10^−73^) and 50% (E-value = 2 × 10^−81^) identity with those of FgDFV1, SlaMFV1, and SsDFV1, respectively.

The SsDFV2 replicase consists of 2114 amino acids, and is larger than SsDFV1 (2078 amino acids); there is an amino acid identity of 446/1185 (38%) and similarity of 625/1185 (52%) between these two viruses (NCBI Blastp). Compared with the majority of closely related viruses, including FgDFV1 and SlaMFV1, the amino acid identities were 441/1159 (38%) and 405/1134 (36%), with similarities of 602/1159 (51%) and 588/1134 (51%), respectively (NCBI Blastp). These data suggest that SsDFV2 differs significantly from previously reported viruses.

Phylogenetic trees based on multiple alignments of the entire replicase, methyl transferase, helicase, or RdRp domains of SsDFV2 with those of other selected viruses from the order Tymovirales were constructed (Figure 5). This phylogenetic analysis revealed that SsDFV2 is closely related to viruses of the family *Deltaflexiviridae*.

### 3.4. SsDFV2 can be Transmitted Among Individuals that Exhibit Vegetative Incompatibility with the Host

**Figure 6:**
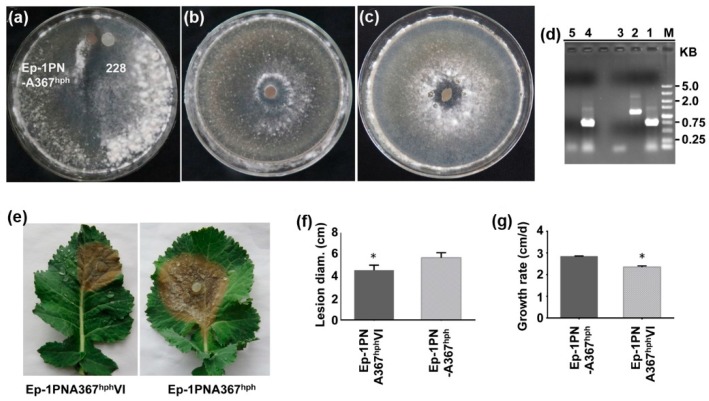
SsDFV2, but not BpRV1, transmitted from *S. sclerotiorum* strain 228 to its vegetative incompatible strain, Ep-1PNA367. (**a**–**c**) Colony morphologies of dual-cultured strains Ep-1PNA367 and 228, and Ep-1PNA367 and virus-infected Ep-1PNA367VI. (**d**) Viral detection by RT-PCR amplification using BpRV1 and SsDFV2 sequence-specific primer pairs. Lane M, DL5000 DNA Marker; Lanes 1,2, SsDFV2 specific band (~750 bp) and BpRV1 specific band (1200 bp) were amplified from strain 228; Lane 3, ddH_2_O (control); Lane 4, SsDFV2 specific band in strain Ep-1PNA367VI; Lane 5, No BpRV1 band in strain Ep-1PNA367VI; (**e**,**f**) Weak virulence of Ep-1PNA367 after virus infection on detached rapeseed leaves at 72 hpi under conditions of 20 °C and 100% relative humidity. (**g**) Decreased growth rate of strain Ep-1PNA367VI. Colonies were grown on PDA for 7 days at 20 °C; significantly different values (*p* < 0.05) are indicated by asterisks.

Most RNA mycoviruses can easily be transmitted among host’s vegetative compatible individuals via hyphal anastomosis, however they cannot transmit with high efficiency among host’s vegetative incompatible individuals. To evaluate the potential transmission of SsDFV2 among host’s vegetative incompatible individuals, strain 228 was dual cultured with it incompatible strain, Ep-PNA367*^hph^*, on PDA plates. After the two colonies intermingled, ten mycelial agar discs were taken from the Ep-PNA367*^hph^* colony and transferred to hygromycin-containing PDA plates for further growth. Next, the newly developed Ep-PNA367*^hph^* colonies were screened for virus by RT-PCR amplification. SsDFV2 was successfully detected in all newly developed colonies, while BpRV1 was not be detected in any of the same colonies (Figure 6, Figure S1). This result suggests that SsDFV2, but not BpRV1, can be transmitted from strain 228 to its vegetative incompatible strain, Ep-1PNA367. A similar experiment was performed using another vegetative incompatible strain, 1980*^hph^*, and yielded consistent results (Figure S2).

**Figure 7:**
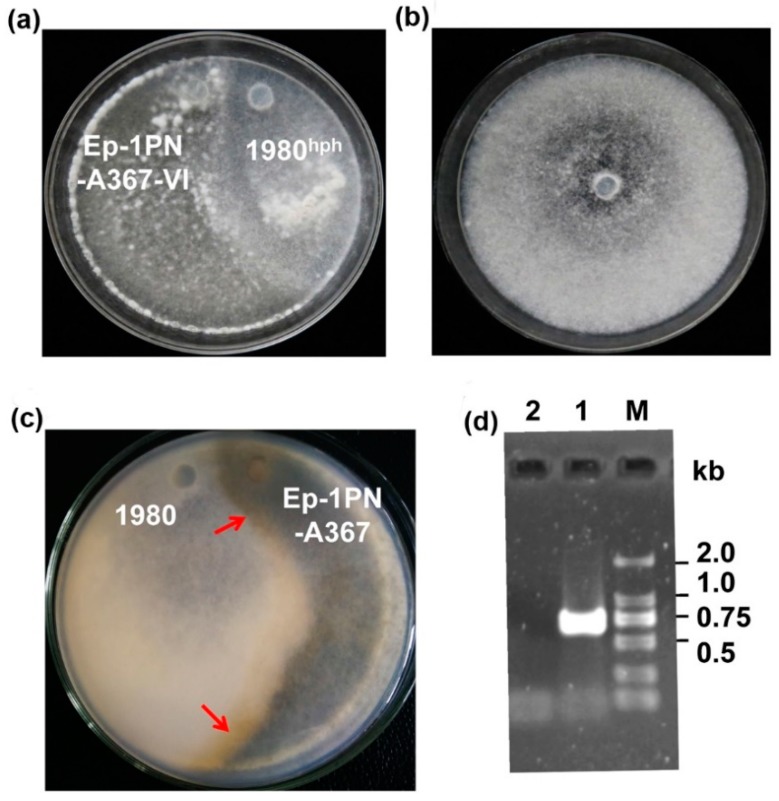
SsDFV2 can be transmitted from *S. sclerotiorum* strain Ep-1PNA367VI to its vegetative incompatible strain, 1980. (**a**) Dual-culture colonies; (**b**) Virus-infected strain (1980) colony; (**c**) Colonies in dual-cultures of strains Ep-1PNA367 and 1980 grown for 7 days, showing the vegetative incompatibility reaction area (red arrow). (**d**) Virus detection in the virus-infected strain, 1980, by RT-PCR amplification using SsDFV2 sequence-specific primers. Lane M, DL2000 DNA Marker; Lane 1, SsDFV2 specific band; Lane 2, ddH_2_O.

To determine whether virus transmission was independent of the host strain (228), the SsDFV2-infected strain, Ep-1PNA367VI, was dual cultured with strain 1980*^hph^*. After the two colonies intermingled, mycelial agar discs were taken from the 1980*^hph^* colony and placed on fresh hygromycin-containing PDA plates for further growth and virus evaluation. SsDFV2 was detected in the newly emerging colony (Figure 7), confirming that this virus can be independently transmitted among vegetative incompatible host individuals.

### 3.5. SsDFV2 Had a Minor Influence on the S. sclerotiorum Phenotype

Strain 228 was originally co-infected with SsDFV2 and BpRV1, which can confer hypovirulence to *Botrytis* sp. Although strain 228 appeared hypovirulent, whether SsDFV2 alone could confer hypovirulence to its host was not known. Therefore, we separated the two viruses by dual culturing strain 228 with strain Ep-1PNA367*^hph^*, and then transmitted SsDFV2 from the recipient Ep-1PNA367*^hph^* strain to strain Ep-1PNA367. The SsDFV2-infected strain, Ep-1PNA367VI, showed a slower growth rate (2.4 cm/day) than the virus-free strain, Ep-1PNA367 (2.81 cm/day), along with delayed sclerotial development. Strain Ep-1PNA367VI also exhibited slightly lower pathogenicity than its virus-free counterpart, evidenced by the smaller lesions (4.5 cm) it produced on fresh rapeseed leaves, whereas the virus-free strain generated larger lesions (5.7 cm) (Figure 6). Thus, SsDFV2 appears to have a minor influence on the virulence and growth of its hosts.

## 4. Discussion

In the present study, we isolated and characterized a novel mycovirus, SsDFV2, from strain 228 of *S. sclerotiorum* using RNA_Seq analysis followed by RACE cloning. According to phylogenetic analysis, SsDFV2 is a member of the order Tymovirales. Recently, the order Tymovirales was divided into five families, *Alphaflexiviridae, Betaflexiviridae, Deltaflexiviridae, Gammaflexiviridae*, and *Tymoviridae* (https://talk.ictvonline.org/taxonomy); based on genomic organization, phylogenetic relationships, and virion morphology [31]. Phylogenetic analysis based on the entire replicase, methyltransferase, helicase, and RdRp sequences of SsDFV2 demonstrated that it is closely related to the viruses SsDFV1, SlaMFV1, and FgDFV1, and could be tentatively assigned to the family *Deltaflexiviridae*.

The genome organization of SsDFV2 is significantly different from other reported viruses in the family *Deltaflexiviridae*. The SsDFV2 has only one gene, which encodes an RNA replicase, while SsDFV1 and SlaMFV1 each have four genes encoding a replicase and three small proteins, and FgDFV1 has five genes [18,23,24]. SsDFV1 is not likely to have a gene encoding a coat protein, although it is not known whether SlaMFV1 and FgDFV1 have coat proteins. In the order Tymovirales, some members of the three families, *Alphaflexiviridae, Deltaflexiviridae*, and *Gammaflexiviridae*, are reported to infect fungi, Botrytis virus X, in the family *Alphaflexiviridae*, has flexuous rod-shaped particles and five genes [32], and Botrytis virus F, in the family *Gammaflexiviridae*, also has flexuous rod-shaped particles, while its genome consists of two genes [33]. *Sclerotinia sclerotiorum* debilitation-associated RNA virus (SsDRV), in the family *Alphaflexiviridae*, does not have any coat protein, and its genome comprises a single gene [34]. Recently, viruses which can shuttle between fungi and plants have been identified [35,36], suggesting that some fungal viruses may originate in plant viruses, and then evolve in fungal cells, losing non-essential genes. This phenomenon may occur widely in nature, because many (+) single-stranded mycoviruses in different families do not have any coat protein gene. For example, in the proposed family *Fusariviridae*, the numbers of genes differ among the members, and all reported fusariviruses lack genes encoding coat proteins [26,37,38,39,40], which is also the case for viruses in the family *Hypoviridae*.

The co-infection of a single fungal strain by multiple mycoviruses is a common phenomenon in nature [41]. Strain W8 of *Rosellinia necatrix* is reported to be infected with multiple viruses [42] and 12 dsRNA segments, revealed to be mitovirus genomes, co-infect *Ophiostoma novo-ulmi* [43,44], while strain GX-1 is reported to contain seven dsRNA fragments [26]. There are numerous examples of mixed infections of *S. sclerotiorum*, including the hypovirulent strains 382, 16235, SZ-150, and Ep-1PN, and there is also one example of a virulent strain, Sunf-M, reported to be co-infected with two or three mycoviruses [15,45,46]. In the present study, deep sequencing and RT-PCR amplification analysis showed that strain 228 contains two mycoviruses. Hence, this report presents another example of the complexity of mycoviruses in the plant pathogenic fungus, *S. sclerotiorum*. A possible reason for the phenomenon of multi-infection of this species by mycoviruses is that *S. sclerotiorum* can form sclerotia, a fungal dormancy structure that can survive for long periods of time in disadvantageous conditions and directly germinate to infectious hyphae for plant invasion. A high frequency of multi-infection was also found in the sclerotial-forming fungi, *Rhizoctonia solani* and *Botrytis cinerea* [47,48,49,50,51]. The interactions between fungi and viruses are well studied [52,53,54,55], and mycoviruses can suppress host antiviral systems [56,57], which may facilitate co-infection.

The transmission of mycoviruses in host populations will be key for the use hypovirulence-associated mycovirus to facilitate control of crop fungal diseases. Mycoviruses may transmit vertically, via fungal spores, or horizontally, via hyphal anastomosis, with very rare exceptions, including the fungal DNA virus SsHADV-1, in the family *Genomoviridae*, which can directly infect host hyphae and has an insect transmission vector [11], and a partitivirus that can even be transmitted among different host species by dual culture [58]. There are several reports indicating that fungal vegetative incompatibility significantly reduces the natural spread of mycoviruses between different fungal strains [4]; however, we recently found that a mycoreovirus (SsMYRV4) could suppress host vegetative incompatibility responses to facilitate the transmission of other viruses and itself among host vegetative incompatible groups [12]. In this study, we found that SsDFV2, but not BpRV1, could be transmitted among host vegetative incompatibility groups by dual culture; and surprisingly, we also observed that BpRV1 even could not be easily transmitted among host vegetative compatibility groups. This finding suggests that, even without viral particles, some (+) ssRNA mycovirus could overcome the vegetative incompatibility system to transmit between host populations. Further investigation of the transmission mechanism of SsDFV2 may provide important clues to understanding the ecology of mycoviruses in nature and may thus assist in further exploration of hypovirulence-associated mycoviruses for use in the control of crop fungal diseases.

## Figures and Tables

**Table 1:** Primers used in this study.

Primer Name	Sequence
SsDFV2-R1	5′-GGGAGGCGATTGACAGG-3′
SsDFV2-F1	5′-TGTCTTTGGCAGTACCCTCC-3′
BpRV1-dsRNA1-R1	5′-AGCCAGGTATGCGTGAATG-3′
BpRV1-dsRNA1-F1	5′-CAAGGGTAAAGGAAGCGACT-3′
BpRV1-dsRNA2-R1	5′-GGCCTCATCCCAGATTAAG-3′
BpRV1-dsRNA2-F1	5′-CTGTCTACTCGGGCTCAAGG-3′
PC3-T7 Loop adapter	5′-p-GGATCCCGGGAATTCGGTAATACGACTCACTATATTTT TATAGTGAGTCGTATTA-OH-3′
PC2 primer	5′-p-CCGAATTCCCGGGATCC-3′
Seq. specific primer—5end SsDFV2-5end-R	5′-CGGGATCATCAATGCGAC-3′
Seq specific primer—3 end SsDFV2-3end-F	5′-TTCAACCACATCCGAATCTT-3′

**Table 2:** The amino acid sequence identity between the methyltransferase, helicase, and RdRp motifs of SsDFV2 and those of selected viruses of the order Tymovirales.

Family	Virus Name	Abbr.	NCBI Accession Number	Mtr (Identity %)	Hel (Identity %)	RdRp (Identity %)
*Alphaflexiviridae*	Potato Virus X	PVX	NC_011620.1	34/205 (17%)	59/238 (25%)	54/237 (23%)
	Yam Virus X	YVX	NC_025252.1	54/263 (20%)	60/235 (26%)	53/225 (24%)
	Cassava virus X	CVX	NC_034375.1	75/296 (25%)	25/156 (16%)	37/225 (16%)
	Sclerotinia sclerotiorum debilitation-associated RNA virus	SsDRV	NC_007415.1	72/263 (27%)	25/108 (23%)	46/201 (23%)
*Tymoviridae*	Okra Mosaic Virus	OMV	NC_009532.1	75/244 (31%)	-	29/129 (22%)
	Grapevine rupestris vein feathering virus	GRVFV	KY513702.1	87/294 (30%)	32/198 (16%)	45/202 (22%)
	Grapevine Syrah Virus 1	GSV	JX513896.1	82/294 (28%)	27/191 (14%)	26/146 (18%)
*Gammaflexi-viridae*	Botrytis Virus F	BVF	NC_002604.1	60/177 (34%)	40/235 (17%)	61/218 (28%)
*Betaflexiviridae*	Citrus leaf blotch virus	CLBV	KR023647.1	44/291 (15%)	24/132 (18%)	75/251 (30%)
	Cherry rusty mottle associated virus	CRMAV	KP258176.1	32/243 (13%)	34/238 (14%)	73/250 (29%)
	Shallot latent virus	SLV	LC279526.1	9/264 (30%)	20/144 (14%)	53/191 (28%)
	Garlic common latent virus	GCLV	KX255694.1	49/291 (17%)	81/257 (32%)	64/262 (24%)
	Blueberry scorch virus	BSV	AY941199.1	75/258 (29%)	30/224 (13%)	77/254 (30%)
	Cowpea mild mottle virus	CMMV	NC_014730.1	73/249 (29%)	-	75/254 (30%)
*Deltaflexiviridae*	Soybean leaf-associated mycoflexivirus 1	SlaMFV1	KT598226.1	110/292 (38%)	101/243 (42%)	122/262 (47%)
	Fusarium graminearum deltaflexivirus 1	FgDFV1	NC_030654.1	111/292 (38%)	113/247 (46%)	134/262 (51%)
	Sclerotinia sclerotiorum deltaflexivirus 1	SsDFV1	KT581451.1	101/292 (35%)	105/244 (43%)	132/262 (50%)
Unlcassified Flexivirus	Rhizoctonia solani flexivirus 1	RsFV1	NC_030655.1	87/234 (37%)	71/157 (45%)	58/196 (29%)
	Rhizoctonia solani flexivirus 2	RsFV2	KX349069.1	-	-	97/262 (37%)

Note: “-” indicates no homology.

## Supplementary Materials

The following are available online at http://www.mdpi.com/1999-4915/10/6/295/s1, Figure S1: Confirmation of viral transmission of SsDFV2, but not BpRV1, from strain 228 to its vegetative incompatible strain Ep-1PNA367, Figure S2: SsDFV2 transmitted from *S. sclerotiorum* strain 228 to its vegetative incompatible strain 1980.
