# Supplementary material for: A Novel Deltaflexivirus that Infects the Plant Fungal Pathogen, Sclerotinia sclerotiorum, Can Be Transmitted Among Host Vegetative Incompatible Strains
Source: Viruses. 2018 May 31;10(6):295. doi: 10.3390/v10060295 (PMC6024712; doi:10.3390/v10060295)
Supplement: Supplementary file 1 [file viruses-10-00295-s001.pdf]

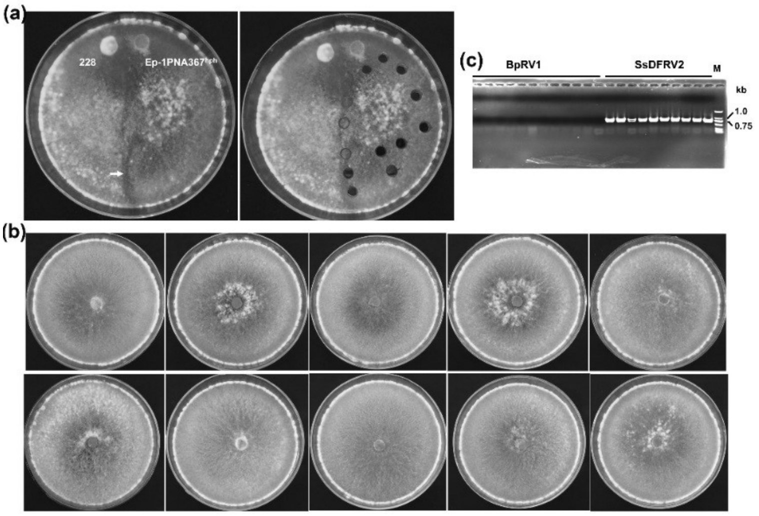

Figure S1. Confirmation of viral transmission of SsDFV2, but not BpRV1, from strain 228 to its vegetative incompatible strain Ep-1PNA367. (**a**) Colonies in dual cultures (7 days on PDA). Colonies were photographed before (left) or after punching (right), showing the locations of each hyphal agar disc. The white arrow shows the vegetative incompatibility reaction. (**b**) Colonies grown from mycelial agar discs on hygromycin-containing PDA. (**c**) Virus detection by RT-PCR amplification using BpRV1 and SsDFV2 sequence specific primer pairs. No BpRV1 specific DNA was amplified. Samples were extracted from ten subcultures.

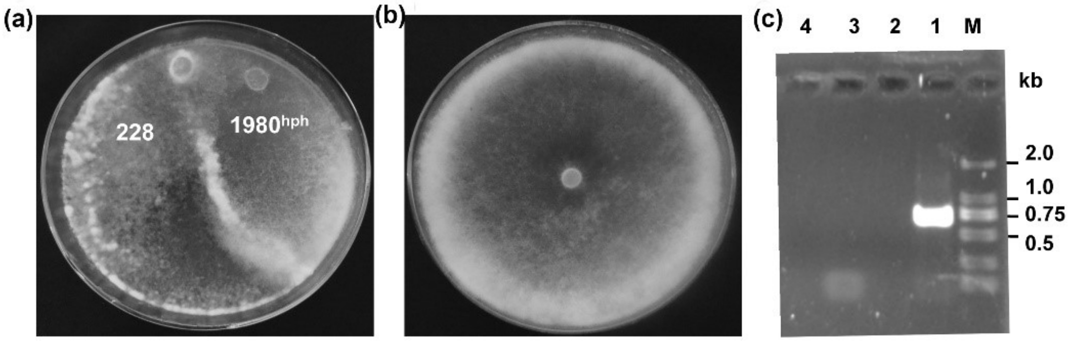

Figure S2. SsDFV2 transmitted from *S. sclerotiorum* strain 228 to its vegetative incompatible strain 1980. (**a**) Dual culture colonies; (**b**) Colony of the virus-infected strain, 1980 (1980VI) (7 days on PDA); (**c**) Virus detection by RT-PCR amplification using BpRV1 and SsDFV2 sequence specific primer pairs. Lane M, DL2000 DNA Marker; Lane 1, SsDFV2 specific band; Lane 2, No band for BpRV1 was amplified; Lane 3, ddH_2O (control with SsDFV2 primers); Lane 4, ddH_2O (control with BpRV1 primers).
